# Teaching and mental health in medical students

**DOI:** 10.1590/1806-9282.20231423

**Published:** 2024-05-13

**Authors:** Halley Ferraro Oliveira, Maria Regina Domingues Azevedo, José Rodrigo Santos Silva, Helena Andrade Figueira, Neusa Falbo Wandalsen, Roseli Oselka Saccardo Sarni

**Affiliations:** 1 Centro Universitário Faculdade de Medicina do ABC – Santo André (SP), Brazil.; 2 Universidade Federal de Sergipe – São Cristóvão (SE), Brazil; 3 Universidade Federal do Rio de Janeiro, Human Motor Bioscience Laboratory – Rio de Janeiro (RJ), Brazil.

**Keywords:** Students, medical, Quality of life, Emotional stress, Depression, Anxiety, Education

## Abstract

**OBJECTIVE:**

The objective of this study was to evaluate the relationship between quality of life, perceived stress, anxiety, and depression in medical students and the university teaching method: traditional method versus active methodology.

**METHODS:**

Four questionnaires were administered to volunteer students (n=361) enrolled in two institutions that employ active (Universidade Tiradentes) or traditional (Faculdade de Medicina do ABC) teaching methodology: socioeconomic level; brief quality of life (World Health Organization Quality of Life-Bref); perceived stress scale (PSS10); and depression and anxiety scale (hospital anxiety and depression scale).

**RESULTS:**

Of the students who responded to the questionnaires (226 UNIT and 135 FMABC), 70% were female and 67% were White. The majority did not use medication for depression (90%), anxiety (81%), and stress management (91%). Regarding anxiety, it was found: absence in the traditional method and moderate anxiety in the active methodology (26% UNIT×13% FMABC) (p<0.001). Regarding quality of life, it was found to be better quality of life in the environment domain at FMABC (78.12%) versus 71.88% at the UNIT (p<0.001). There was no difference between the institutions in relation to depression and perceived stress, and in quality of lifethere was only a difference in the environmental domain (p<0.001). In relation to gender, stress was higher in females (93.7%) than males (79.6%) with p<0.001.

**CONCLUSION:**

Differences were recorded between the groups regarding anxiety, with a predominance in UNIT students (active methodology), and no differences were recorded in relation to depression, perceived stress, and quality of life in all domains, except for the environment domain, which was higher in the traditional methodology, although about one-third of participants used medication for anxiety/depression.

## INTRODUCTION

Concerns about the mental health of university students have become relevant because studies have shown a high occurrence of psychological and psychiatric disorders in the general population. It is estimated that 15–25% of university students have psychiatric disorders, with depression and anxiety being the most common among medical students, whose rates of depression and suicidal ideation are higher than in the general population^1-3^.

Quality of life is a complex and subjective concept, influenced by the individual's physical health, psychological state, level of independence, living conditions, and social relationships^4^. Stress corresponds to a series of neuroendocrinological changes suffered by an individual, called as "general adaptation syndrome," resulting from sources internal or external to the organism^5^.

Anxiety is defined as feelings of apprehension, anguish, worry, and intense restlessness and may lead to systemic cardiorespiratory changes and oppression^6^. Depression is characterized as a mental disorder that determines changes in mood or affect, with functional disability and decreased quality of life^7^.

In Brazil, two teaching methodologies are adopted in medical schools: traditional, where teaching is based on the student/teacher approach, and active, when the student is the center and responsible for building their learning. Several publications address aspects related to mental health and quality of life in medical students. However, there is a gap in the approach to these aspects in relation to teaching methods, justifying this study.

## METHODS

After approval by the FMABC Ethics Committee (CAE no. 13152119.2.0000.0082), a cross-sectional, observational study was carried out with a quantitative approach, with analysis of self-administered questionnaires answered online by medical students of both genders who aged at least 18 years and enrolled in the second, third and fourth years of the course, constituting two groups, according to the pedagogical political plan of each institution, both private: GROUP 1: UNIT (UNIT)—Aracaju, SE, active methodology of teaching and GROUP 2: FMABC (FMABC)—Santo André, SP, traditional teaching methodology. Students in the first, fifth, and sixth years were excluded, due to the additional stress they are subjected to (entering college, change in course dynamics, social environment, being a better professional, studying for the residency exam, and finding a job, among others). The recruitment and data collection period was between April and August 2021. Inclusion criteria were medical students, over the age of 18 years, of both sexes, enrolled in the second, third, and fourth years of the course.

The following questionnaires were used: (1) demographic and socioeconomic issues to characterize the sample; (2) WHOQOL questionnaire—abbreviated, the WHOQOL-Brief, (3) perceived stress scale (PSS-10); and (4) anxiety and depression scale (hospital anxiety and depression scale—HADS). All questionnaires used were adapted, translated, and validated for the Brazilian population. For the sample calculation, the Barbetta formula was used (95% reliability).

Of the 490 enrolled at UNIT and 290 at FMABC, excluding students in the first, fifth, and sixth years, 361 questionnaires were completed appropriately, with 226 from UNIT and 135 from FMABC, after signing the consent form. The exploratory analysis was carried out by calculating simple frequency and percentage for qualitative variables, and median and minimum and maximum interquartile range for quantitative variables, using a Microsoft Excel spreadsheet, and statistical analyses were carried out in the R software, version 4.1.0 (The R Core Team, 2021). The significance level adopted was 5%.

## RESULTS

Of the total of 361 students (226 UNIT and 135 FMABC) who responded to the survey, 70% were female, aged between 18 and 39 years (mean=22 years), White (92.2% FMABC, 52.2% UNIT), Niger (0.7% FMABC, 8% UNIT), and Brown (1.5% FMABC, 37.6% UNIT). The majority declared themselves to be Catholic (30% FMABC, 54.4% UNIT), to have no religion (51.1% FMABC), to live at home with their parents (65.6% FMABC, 64.2% UNIT), to share house in shares home with one or more colleagues (19.3% FMABC, 12.4% UNIT), and to stay alone (11.9% FMABC, 16.4% UNIT).

Most students lived less than 30 min from the institution (UNIT—90.7% versus FMABC—40.4%, p<0.001), the car was the most used means of transport to college (74% FMABC, 66.4% UNIT) (p=0.116), and 27% had student financing at UNIT versus 10.4% at FMABC (p<0.001).

Table 1 shows the percentage of students who used medication for health and for mental disorders and stress. It was found that 36.3 and 34.9% of FMABC and UNIT students used medication, respectively.

**Table 1:** Frequency of medication consumption among medical students at two private educational institutions (FMABC and UNIT) (n=361).

Variables		FMABC	UNIT	p-value
		135 (%)	226 (%)	
Use of medicines for health	No	84 (62.2)	157 (69.5)	0.194^1^
	Yes	51 (37.8)	69 (30.5)	0.194^1^
Use of medicines for depression	No	115 (85.2)	211 (93.4)	<0.001^1^
	Yes	20 (14.8)	15 (6.6)	<0.001^1^
Use of medicines for anxiety	No	109 (80.7)	184 (81.4)	0.984^1^
	Yes	20 (19.3)	42 (18.6)	0.984^1^
Use of medicines for stress control	No	126 (93.3)	204 (90.3)	0.416^1^
	Yes	9 (6.7)	22 (9.7)	0.416^1^

1 Significance level of the chi-square test.

All FMABC students had stress and, at the UNIT, 96% of them reported some degree of stress, but there was no statistical difference. In relation to gender, stress (moderate and high) was higher in females (93.7%) than males (79.6%) with p<0.001. Anxiety was reported with p<0.001: absence, in the traditional method (51.1%) versus (45.1%) in UNIT; more frequent moderate anxiety, in the active methodology (26.5%) versus traditional method (13.3%). On the contrary, depression was mostly not reported (70.4% FMABC and 76.5% UNIT) and the other degrees (mild, moderate, and severe) with no statistical difference in both HEIs, as we can observe in Table 2.

**Table 2:** Responses to the perceived stress scale and HDA questionnaires by medical students from two private institutions (FMABC and UNIT) (n=361).

Variables		FMABC	UNIT	p-value
		135 (%)	226 (%)	
PSS-10 questionnaire				
	Low	17 (12.6)	21 (9.3)	0.511^1^
(Perceived stress)				
	Moderate	72 (53.3)	118 (52.2)	0.511^1^
	High	46 (34.1)	87 (38.5)	0.511^1^
HAD-A questionnaire				
	No	65 (51.1)	102 (45.1)	<0.001^1^
(Anxiety)				
	Mild	27 (20)	37 (16.4)	<0.001^1^
	Moderate	18 (13.3)	60 (26.5)	<0.001^1^
	Severe	21 (15.6)	27 (11.9)	<0.001^1^
HAD-D questionnaire				
	No	95 (70.4)	173 (76.5)	0.246^2^
(Depression)				
	Mild	18 (13.3)	32 (14.2)	0.246^2^
	Moderate	19 (14.1)	19 (8.4)	0.246^2^
	Severe	3 (2.2)	2 (0.9)	0.246^2^

1 Significance level of the chi-square test.

2 Significance level of the Fisher's exact test.

In relation to the year of teaching, there were no statistical differences in the population studied. However, the second year presented a low risk of stress on average (5.5%) compared with the fourth year (13%). Anxiety in the form of mild or moderate was higher in the second year (46.5%) compared with the fourth year (36.2%), without statistical differences. It was observed that there was a higher risk of mild depression in the second year (18.1%) and fourth year (13%) of teaching and a moderate risk for depression in 9% of students in the second year compared with those in the fourth year (12%), but without statistical difference.

Table 3 shows the results of the questionnaires applied to identify stress, anxiety, depression, and QoL. Statistical differences were recorded regarding the anxiety aspect: absence in FMABC, more frequent moderate anxiety in UNIT (26% UNIT×13% FMABC; p<0.001), and better QoL in the environment domain at FMABC [78.12 vs. 71.88% UNIT (p<0.001)].

**Table 3:** Responses to the WHOQOL and its domains by medical students enrolled in two private educational institutions (FMABC and UNIT) (n=361).

Variables	FMABC	UNIT	p-value
	135 (%)	226 (%)	
Age	22	22	0.957^1^
Domain 1—physical domain	67.86	67.86	0.81^1^
Domain 2—psychological domain	58.33	62.5	0.378^1^
Domain 3—social relations	66.67	66.67	0.691^1^
Domain 4—environment	78.12	71.88	**<0.001** ^1^

1 Significance level of the Mann-Whitney test. The statistically significant p-value is indicated in bold.

## DISCUSSION

The analysis of the QoL questionnaires showed that students from both schools did not differ in the different domains, except the environment, which could be justified by the difference in the Human Development Index (HDI) between the cities (0.815—Santo André and 0.79—Aracaju)^8^. The lowest values occurred in the psychological domain, in agreement with the results of Jesus et al^9^. In females, QL levels were lower in both groups, which is repeated in the literature consulted^10^.

All students reported some degree of stress, with the sum of moderate and severe levels being high (87.4% FMABC and 90.7% Unit). During the course, stress increases due to the demands of academic life and interpersonal and emotional conflicts, such as situations of death and suffering experienced, especially in recent years, during the internship period, when students maintain direct and continuous contact with patients^11,12^. In this study, it was observed that advancing the course increased stress and reduced anxiety, coinciding with the literature consulted^13-16^. The need to improve cognitive performance, concentration, and memory and to increase study time, experimentation, and the influence of friends are factors that can lead to an increase in the use of psychotropic drugs by students^17^.

The prevalence of depression in medical students ranges from 5.60 to 45.70%^18^, and almost 30% of these use some medication for anxiety or depression^13^.

The medical course causes stress, due to students being subjected to fear of failure, self-demand, demands from parents, extensive workload, curricular and extracurricular activities at the college, and situations capable of causing greater susceptibility to various psychiatric disorders^14^. All participants in this research reported some degree of stress, with no difference between the groups, a result greater than that reported in the literature (stress levels ranging from 40.95 to 84.30%) but higher in females, coinciding with the values of this research^15,16^.

In this study, some degree of anxiety was observed in the institution that adopts the traditional methodology and a greater degree in the active methodology. A possible explanation for the greater tendency toward anxiety in individuals who use the active methodology would be the fact that, in this, the student needs to go in search of knowledge. Several authors report a higher frequency of stress, anxiety, and depression in students using the active methodology, when compared with the traditional method, due to the pace imposed by the former, with the need for greater active participation by the student in research, meetings, and discussions^19,20^. A direct relationship between stress and depression has been found in several studies, with a high degree of stress being related to moderate and severe depression, in agreement with other authors^21-23^.

## CONCLUSION

The differences observed between the groups point to a higher frequency of anxiety in students undergoing the active methodology than in the traditional one, with no differences regarding depression and perceived stress. One-third of the participants used medication for anxiety/depression and the score obtained in the psychological domain was low in both universities, suggesting possible psychological distress in students. Monitoring mental health and QoL in medical students is necessary regardless of the teaching method.

## References

[1] Bassols AM, Okabayashi LS, Silva AB, Carneiro BB, Feijó F, Guimarães GC (2014). First- and last-year medical students: is there a difference in the prevalence and intensity of anxiety and depressive symptoms?. Braz J Psychiatry.

[2] Al-Maashani M, Al-Balushi N, Al-Alawi M, Mirza H, Al-Huseini S, Al-Balushi M (2020). Prevalence and correlates of depressive symptoms among medical students: a cross-sectional single-centre study. East Asian Arch Psychiatry.

[3] Bert F, Lo Moro G, Corradi A, Acampora A, Agodi A, Brunelli L (2020). Prevalence of depressive symptoms among Italian medical students: the multicentre cross-sectional "PRIMES" study. PLoS One.

[4] Dias AF, Junior CSC, Moreira VES (2018). Stress and academic performance in students of UNEC Anais do VIII Encontro de Iniciação Científica - ENIC UNEC 2018 - Caratinga-MG.

[5] Schönhofen FDL, Neiva-Silva L, Almeida RBD, Vieira MECD, Demenech LM (2020). Generalized anxiety disorder among pre-university students. J Bras Psi.

[6] Silva NR, Bolsoni-silva AT, Loureiro SR (2018). Burnout and depression in elementary school teachers: a correlational study. Rev Bras Educ.

[7] Moreira EEP, Oliveira SMC, Oliveira JVP (2020). Contributes and challenges of problem-based learning in medical training. Res Soc Dev.

[8] IBGE (2020). Cities.

[9] Jesus AMV, Oliveira HF, Azevedo MRD, Santos RGA, Nogueira HG (2022). Analysis of medical student's life quality: a systematic review. Res Soc Dev.

[10] Silveira DJ, Bomfim R, Silveira KM, Santos EL, Ferro PM, Pimentel D (2021). Mental disorders and academic impact on medical students subjected to the problem-based learning method. Braz J Dev.

[11] Luna IS, Dominato AAG, Ferrari F, Costa AL, Pires AC, Silva Ximendes G (2018). Consumption of psychotropic drugs among first and sixth year medical students at a university in the state of São Paulo. Colloq Vitae.

[12] Cruz PE, Caramona M, Guerreiro MP (2015). A reflection on self-medication and non-prescription medicines in Portugal. Rev Port Farmacoter.

[13] Brito JR, Silva PR (2020). Consumption of anxiolytics and antidepressants: an analysis of their use among medical students.

[14] Silveira DJAS, Bomfim R, Silveira KMAS, Santos EL, Ferro PM, Pimentel D (2021). Mental disorders and the academic impact on medical students submitted to the problem-based learningmethod. Braz J Dev.

[15] Aragão JA, Freire MRM, Nolasco Farias LG, Diniz SS, Sant'anna Aragão FM, Sant'anna Aragão IC (2018). Prevalence of depressive symptoms among medical students taught using problem-based learning versus traditional methods. Int J Psychiatry Clin Pract.

[16] Machado SLM, Sirico NDS, Barbosa PF, Rosa RRM (2019). Anxiety and depression in medical students.

[17] Medeiros JM, Barbosa AG (2019). Nonprescription use of methylphenidate hydrochloride among college students. Rev Int.

[18] Mendes TC, Dias ACP (2021). Symptoms of depression, anxiety, stress and associated factors in Brazilian medical students: integrative review. Res Soc Dev.

[19] Ferreira JL, Rossi SV, Araujo KF, Klein MP, Miranda NR, Campos VR (2016). Prevalence of common mental disorders and associated factors in medical students: a comparative study. Rev Bras Educ Med.

[20] Ariño DO, Bardagi MP (2018). Relationship between academic factors and the mental health of university students. Psicol Pesq.

[21] Ribeiro CF, Lemos CMC, Alt NN, Marins RLT, Corbiceiro WCH, Nascimento MI (2020). Prevalence of and factors associated with depressionand anxiety in Brazilian medical students. Rev Bras Educ Med.

[22] Costa DS, Medeiros NSB, Cordeiro RA, Frutuoso ES, Lopes JM, Moreira SNT (2020). Symptoms of depression, anxiety and stress in medical students and institutional coping strategies. Rev Bras Educ Med.

[23] Solis AC, Lotufo F (2019). Predictors of quality of life in Brazilian medical students: a systematic review and meta-analysis. Braz J Psychiatry.

